# In Vitro and In Vivo Evaluation of Hydroxypropyl-β-cyclodextrin-grafted-poly(acrylic acid)/poly(vinyl pyrrolidone) Semi-Interpenetrating Matrices of Dexamethasone Sodium Phosphate

**DOI:** 10.3390/ph15111399

**Published:** 2022-11-14

**Authors:** Nyla Ajaz, Anum Abbas, Rabia Afshan, Muhammad Irfan, Syed Haroon Khalid, Sajid Asghar, Muhammad Usman Munir, Waleed Y. Rizg, Kamlah Ali Majrashi, Sameer Alshehri, Mohammed Alissa, Mohammed Majrashi, Deena M. Bukhary, Ghulam Hussain, Fauzia Rehman, Ikram Ullah Khan

**Affiliations:** 1 Department of Pharmaceutics, Faculty of Pharmaceutical Sciences, Government College University Faisalabad, Faisalabad 38000, Pakistan; 2 Foundation University Medical College, Islamabad 44000, Pakistan; 3 Women Medical College, Abbottabad 22020, Pakistan; 4 Department of Pharmaceutical Chemistry, College of Pharmacy, Jouf University, Sakaka 72388, Saudi Arabia; 5 Department of Pharmaceutics, Faculty of Pharmacy, King Abdulaziz University, Jeddah 21589, Saudi Arabia; 6 Biological Sciences Department, College of Science & Arts, King Abdulaziz University, Rabigh 21911, Saudi Arabia; 7 Department of Pharmaceutics and Industrial Pharmacy, College of Pharmacy, Taif University, P.O. Box 11099, Taif 21944, Saudi Arabia; 8 Department of Medical Laboratory Sciences, College of Applied Medical Sciences, Prince Sattam bin Abdulaziz University, Al-Kharj 11942, Saudi Arabia; 9 Department of Pharmacology, College of Medicine, University of Jeddah, Jeddah 23890, Saudi Arabia; 10 Department of Pharmaceutics, College of Pharmacy, Umm Al-Qura University, Makkah 24211, Saudi Arabia; 11 Department of Physiology, Government College University Faisalabad, Faisalabad 38000, Pakistan

**Keywords:** sustainability of natural resources, semi-interpenetrating, pH-sensitive networks, acrylic acid, poly(vinyl pyrrolidone), hydroxypropyl-β-cyclodextrin, dexamethasone

## Abstract

In this paper, we fabricated semi-interpenetrating polymeric network (semi-IPN) of hydroxypropyl-β-cyclodextrin-*grafted*-poly(acrylic acid)/poly(vinyl pyrrolidone) (HP-β-CD-g-poly(AA)/PVP) by the free radical polymerization technique, intended for colon specific release of dexamethasone sodium phosphate (DSP). Different proportions of polyvinyl pyrrolidone (PVP), acrylic acid (AA), and hydroxypropyl-beta-cyclodextrin (HP-β-CD) were reacted along with ammonium persulphate (APS) as initiator and methylene-bis-acrylamide (MBA) as crosslinker to develop a hydrogel system with optimum swelling at distal intestinal pH. Initially, all formulations were screened for swelling behavior and AP-8 was chosen as optimum formulation. This formulation was capable of releasing a small amount of drug at acidic pH (1.2), while a maximum amount of drug was released at colonic pH (7.4) by the non-Fickian diffusion mechanism. Fourier transformed infrared spectroscopy (FTIR) revealed successful grafting of components and development of semi-IPN structure without any interaction with DSP. Thermogravimetric analysis (TGA) confirmed the thermal stability of developed semi-IPN. X-ray diffraction (XRD) revealed reduction in crystallinity of DSP upon loading in the hydrogel. The scanning electron microscopic (SEM) images revealed a rough and porous hydrogel surface. The toxicological evaluation of semi-IPN hydrogels confirmed their bio-safety and hemocompatibility. Therefore, the prepared hydrogels were pH sensitive, biocompatible, showed good swelling, mechanical properties, and were efficient in releasing the drug in the colonic environment. Therefore, AP-8 can be deemed as a potential carrier for targeted delivery of DSP to treat inflammatory bowel diseases.

## 1. Introduction

Hydrogels are three-dimensional crosslinked polymeric structures with an excellent capability to hold water and biological fluids within the network [1,2]. In the last two decades, they have gained remarkable reputation as drug carriers for various applications owing to their remarkable features. They can act as a stimuli-sensitive drug delivery system by responding to various stimuli such as pressure, pH, ionic strength, and temperature [3,4,5]. However, low mechanical strength, difficulty in drug loading, and premature drug release are the few drawbacks associated with simple hydrogels. These shortcomings can be overcome by using the interpenetrating polymer network (IPN). IPN is an admixture of two or more crosslinked polymers, noticeably one of which is synthesized or crosslinked in the instant presence of other polymer without any covalent bond between polymer chains and cannot be separated unless chemical bonds are broken [6]. When a linear polymer is entrapped in the matrix, it is labeled as semi-IPN [7]. Semi-IPN hydrogels are composed of synthetic or natural polymers and are significantly better than simple hydrogels in terms of mechanical and swelling properties [8]. They not only improve drug diffusion and loading, but also impart mechanical strength when equated with conventional hydrogels [9]. Moreover, the stimuli-sensitive behavior of semi-IPN hydrogels have been developed for numerous applications in drug delivery and biomedicine [10,11].

PVP is a hydrophilic, nontoxic, biocompatible, and biodegradable synthetic polymer, which makes it a suitable contender with other polymers for synthesizing IPN or semi-IPN hydrogels [12]. Individually, it has an appreciable mechanical strength but inferior swelling characteristic, which can be improved by copolymerization with a suitable monomer, such as vinyl monomers, AA or methacrylate [3]. Previously, PVP had been used along with carboxymethylcellulose [13], sodium alginate [14], poly(vinyl alcohol) [15], chitosan [16], etc. for hydrogel-based carriers for delivery of numerous active ingredients and biomedical applications.

AA contains carboxylic (–COOH) [17,18], which makes it a suitable candidate for the development of hydrogels, which are sensitive to ionic strength and pH of media [19]. AA had been used with gelatin [20], PVP [3], vinylsulfonic acid [4], chitosan [21], guar gum [22], poly(vinyl alcohol) [23], etc. to develop pH-sensitive hydrogels.

Cyclodextrins (CDs) are torus-like oligosaccharides comprising of six to eight glucopyranose units linked by α-1,4-glycosidic bonds and are known as α, β, and γ-cyclodextrin [24]. CDs make inclusion complexes with organic molecules through host-guest interactions [25]. The efficiency of CDs can be increased by incorporating them with an insoluble three-dimensional polymeric structure [26]. In these cases, attached cyclodextrins form complexes with hydrophobic drugs of suitable size or, in the case of hydrophilic drugs, form complexes with an accessible hydrophobic portion [27]. Moreover, the addition of CDs to the polymer matrix structure provides (i) an affinity-based mechanism of drug loading and controlled drug release, and (ii) enhanced hydrophilicity of polymer matrix.

Among various CDs, HP-β-CD is highly successful in promoting the dissolution of water insoluble active ingredients [28]. It has strong absorption and solubilizing power with maximum stability and minimum toxicity [29]. Furthermore, it increases the bioavailability of complex compounds [30] and forms complexes with several hydrophilic polymers, such as PVP [31], hydroxypropyl methylcellulose (HPMC), and polyethylene glycol (PEG) [32] to modify physiochemical properties, improve stability, biocompatibility, drug loading, and drug release. Therefore, this makes it useful for various drug delivery applications [33,34]. The presence of HP-β-CD in free or covalently-linked IPN hydrogels improves the release rate of entrapped drug by enhancing the dissolution and diffusion of the drug [35].

Dexamethasone sodium phosphate (DSP) is a water-soluble anti-inflammatory derivative of dexamethasone [36]. It has been extensively used to treat colonic diseases, such as bowel diseases, ulcerative colitis [37], Crohn’s disease, amoebiasis, colonic cancer, etc.

In literature, numerous hydrogels were developed for drug delivery applications using the above-mentioned components in various combinations. For example, PVP/AA with tragacanth gum [38], PVP/poly(ethyleneglycol)-dimethacrylate with CD [35], PVP-poly(ethylene glycol) containing inclusion complexes of ibuprofen in β-cyclodextrin [39], as well as pH-responsive hydrogel based on guar gum, poly(acrylic acid), and β-cyclodextrin [40]. To the best of our knowledge, AA, PVP, and HP-β-CD have never been blended to develop the semi-IPN hydrogel.

In the present work, we emphasized on the design of an optimized vehicle for loading and delivery of DSP to colonic region. The synthesized pH-sensitive semi-IPN matrices were characterized by several in vitro assessments and in vivo toxicological tests for possible colonic delivery of DSP to treat ulcerative colitis and Crohn’s disease.

## 2. Results and Discussion

**Figure 1:**
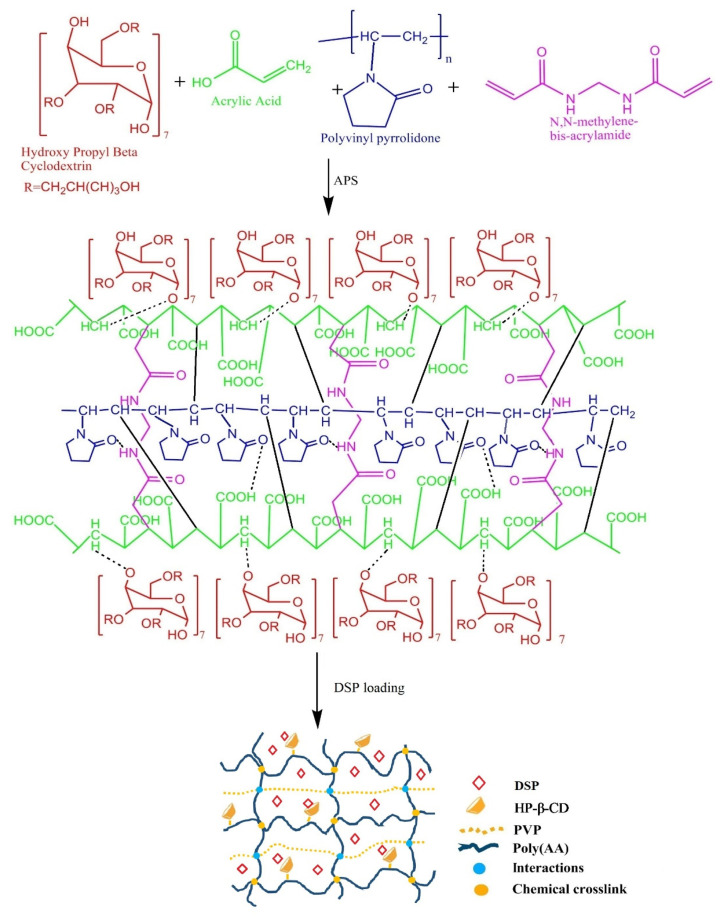
Initial ingredients, synthesis, possible structure, and DSP loading in pH-sensitive semi-IPN hydrogels.

Initially, we developed eighteen semi-IPN hydrogel formulations with variations of AA and PVP (Table 1), and a possible structure is shown in Figure 1. All these formulations were tested for sol-gel behavior and swelling behavior as a function of pH. Concentrations of AA and PVP were varied to select the optimum formulation for further testing.

### 2.1. Swelling Studies

Swelling is an intrinsic property of hydrogels, whereby they are enlarged due to liquid penetration and retention in void spaces among polymeric chains. This property directly affects drug loading and release characteristics of hydrogels. Swelling of hydrogels are affected by pH of media, presence of pH-sensitive groups, interpenetrating polymer, and crosslinking density [5]. As the crosslinker concentration remained constant, we assessed the effect of concentrations of PVP and AA, as well as variations of pH on the swelling behavior of hydrogels as discussed below.

#### 2.1.1. The pH of Medium

**Figure 2:**
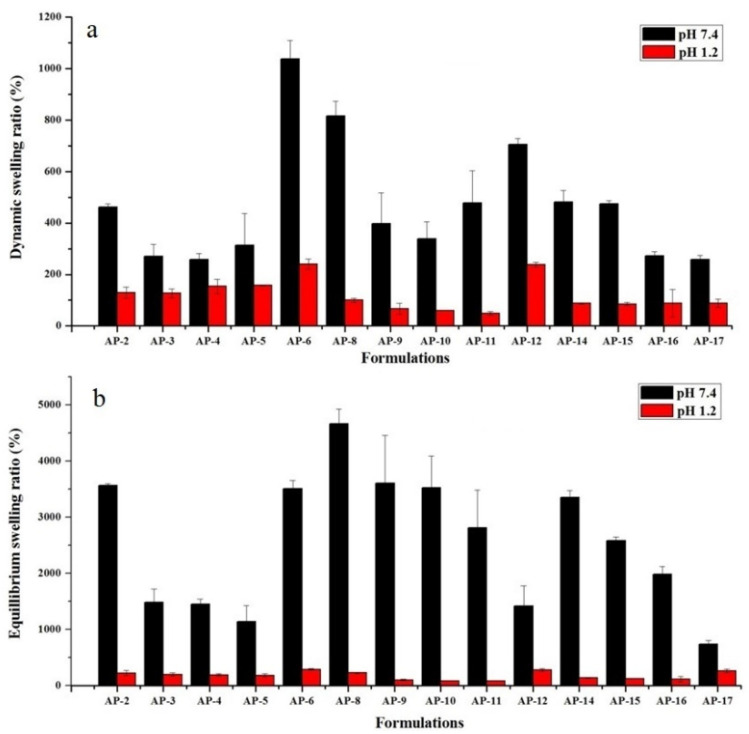
Effect of pH on dynamic swelling ratio (%) at 8 h (**a**) and equilibrium swelling ratio (%) (**b**) of semi-IPN hydrogels. Error bars show standard deviation (*n* = 3). No gel was formed in AP-1, AP-7, and AP-13.

The swelling study determines the time-dependent swelling behavior of semi-IPN hydrogels. The pH of medium significantly affects the physical properties and swelling kinetics of hydrogels. At pH 1.2, hydrogels exhibited a low swelling ratio, when equated with swelling at pH 7.4 as depicted in Figure 2.

**Figure 3:**
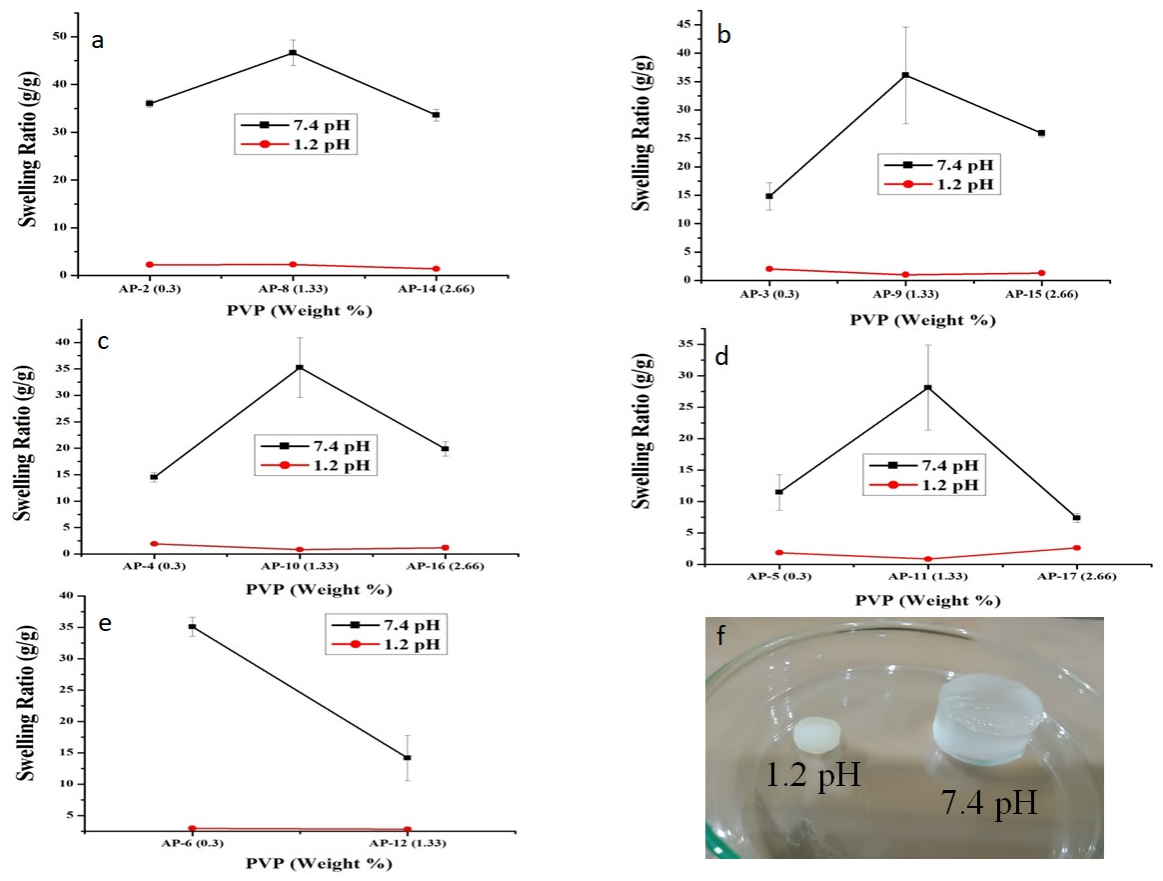
Impact of PVP concentration on equilibrium swelling ratio (g/g) at pH values of 1.2 and 7.4. HP-β-CD remained constant while varying the concentrations of AA, i.e., (**a**) 16.66 g, (**b**) 33.33 g, (**c**) 50 g, (**d**) 66.66 g, and (**e**) 83.33 g. (**f**) Physical appearance of AP-8 at pH values of 1.2 and 7.4. Error bars represent standard deviation (*n* = 3).

Swelling of semi-IPN hydrogels is affected by the presence of carboxylic group (–COOH) of AA. At low pH, hydrogels remained unionized owing to the protonation of –COOH group, which resulted in collapsed hydrogels, thus minimal to no swelling was observed. However, when hydrogels were dipped in simulated intestinal pH, a dramatic surge in swelling ratio was witnessed as shown in Figure 2. This was due to the pH of the surrounding medium, which was above the pKa of AA (4.28) [41]. At this pH, carboxylic groups are easily deprotonated, which results in an increase in electrostatic repulsion and swelling of polymeric network. This difference in the swelling behavior of hydrogels at two different pH values can be visualized in Figure 3f. Moreover, previous studies support our observations. For e.g., Khan and Anwar developed pH-sensitive IPN hydrogels and observed higher swelling at higher pH (7.4) and vice versa [22].

#### 2.1.2. Effect of AA

**Figure 4:**
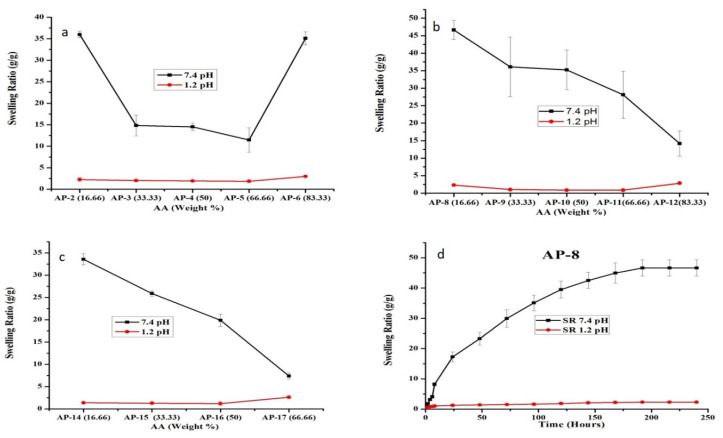
Influence of AA concentration on the equilibrium swelling ratio (g/g) at pH values of 1.2 and 7.4. HP-β-CD concentration remained constant at various concentrations of PVP, i.e., (**a**) 0.3 g, (**b**) 1.33 g, (**c**) 2.66 g. (**d**) Swelling ratio of AP-8 at pH values of 1.2 and 7.4. Here, error bars demonstrate standard deviation (*n* = 3).

After confirming the influence of pH on semi-IPN hydrogels swelling, we explored the swelling behavior with respect to monomer concentration. Here, the swelling ratio declined with the increasing concentration of AA from 16.66 to 66.66 weight % as depicted in Figure 4a–c.

This behavior could stem from the fact that AA is a small entity and hydrogels synthesized with a low AA concentration have greater room for penetration of buffer. In comparison, higher AA content leads to a compact structure that restrains the expansion of network leading to a lesser accessibility of the solvent molecules, thereby exhibiting reduced swelling [22,42]. Our results are in close agreement with the previous studies highlighting decreased swelling with an increasing AA concentration in semi-IPN hydrogels [43,44]. Moreover, as observed in Figure 4a, AP-6 with maximum AA (83.33 weight %) content displayed an abrupt increase in equilibrium swelling (35.08 g/g) at 48 h. At high AA content, low molecular weight polymers are produced by popcorn polymerization that compromise the gel strength leading to rapid swelling as previously observed by Huang et al. [45]. Furthermore, AP-6 was weak and broke easily when immersed in water with a spongy surface. It was sticky after swelling and thus deemed unacceptable. When we compared all formulations, AP-8 showed maximum equilibrium swelling with excellent physical appearance (Figure 4b,d).

#### 2.1.3. Effect of PVP

With the increasing concentration of PVP from 0.3–1.33 weight %, the swelling of hydrogels also increased. This could be described by the following facts. (a) Incorporation of non-ionic PVP increases non-ionic groups in the polymeric matrix. However, it is reported that the collaborative fluid absorption effect of –C=O(N), –COOH, and –COO^–^ groups is significantly better than the individual groups. (b) PVP acts as a dispersant during the reaction to form a consistent network. (c) Hydrophobic alkyl polymeric chain ends of PVP develop tiny hydrophobic regions that facilitate the formation of regular polymeric network [14], which increases the absorption of media. When the PVP content was further increased from 1.33 to 2.66 weight %, polymeric chains were tangled in the network to promote physical crosslinking. This led to the decreased expansion of polymeric network and reduced swelling as observed in Figure 3. Another possible reason could be that after achieving the optimum pore size, further increase in PVP concentration could increase the crosslinking density and restrict the penetration of buffer medium into the polymeric network [46]. Consequently, a decline in hydrogel swelling was witnessed (Figure 3). Similar observations were reported by Wang et al. who developed pH-responsive semi-IPN carboxy methyl cellulose (CMC)-g-poly sodium acrylate/PVP hydrogels. They witnessed increased swelling up to 15 wt%, which decreased with further increase in the concentration of PVP [13]. Similarly, Singh and Sharma also reported a similar effect of PVP on the swelling behavior of PVP-co-poly(2-acrylamido-2-methylpropane sulfonic acid) hydrogels [46]. Initially, swelling increased with the increasing PVP up to 4% and then decreased with further increase in PVP content. Therefore, we can infer that a low concentration of PVP in hydrogel is suitable for achieving optimum strength and swelling. At higher concentrations, PVP not only develops structures that are more resilient, but also increases crosslinking density and reduces pore dimension, which restrict the ingression of dissolution media and swelling.

### 2.2. Sol-Gel Fraction

Sol-gel fraction helps in the estimation of the soluble and insoluble fraction of semi-IPN matrices. Here, a non-crosslinked or soluble portion is referred as a sol, while a crosslinked or insoluble portion is referred as gel fraction hydrogel. Therefore, it helps in the determination of the extent of monomer or polymer utilized for the synthesis of hydrogels [2]. Table 2 demonstrates that increasing the concentration of monomer inversely affects the gel fraction while directly affecting the sol fraction in semi-IPN hydrogels, except for AP-6 as discussed in Section 2.1.2. Similar findings were observed by Abad et al., where the gel fraction decreased with the increasing monomer content for PVP and kappa-carrageenan-based hydrogels [47]. Our results indicate that most of the reactants were available for synthesis of hydrogels under test conditions. Moreover, a small portion of unreacted reactants can be easily extracted as a sol part during washing of hydrogels.

It is noteworthy that the AP-8 formulation had the highest gel fraction and displayed maximum equilibrium swelling when compared with the rest of the formulations. Consequently, a low concentration of AA and intermediate concentration of PVP provided superior properties than its counterpart formulations.

### 2.3. Solid-State Characterization

Solid-state characterization of hydrogels can not only help in the elucidation of network, but it can also predict the purity and stability of formulation. Based on swelling and sol-gel data, AP-8 was chosen as an optimized formulation and deemed suitable for further studies.

#### 2.3.1. FTIR

**Figure 5:**
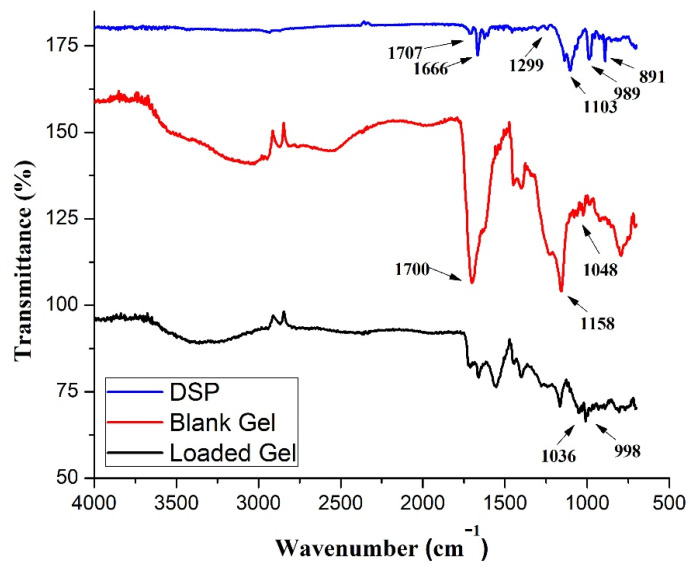
FTIR spectrums of DSP, blank, and drug-loaded semi-IPN hydrogels (AP-8). Blank refers to AP-8 semi-IPN hydrogels without DSP.

FTIR is an easy-to-use, fast, sensitive, and nondestructive analytical technique for pharmaceutical analysis and quality control. It is equally helpful in the identification of functional groups in polymers, plastics, resins, etc. Therefore, we can use this technique for all phases of the pharmaceutical product lifecycle, i.e., design, manufacture, quality control, and failure analysis. In the current research, we employed this technique to confirm the structure of semi-IPN hydrogels and rule out any possible interaction between the loaded drug and hydrogel components. For this purpose, we analyzed the FTIR spectra of DSP, blank, and DSP-loaded semi-IPN hydrogels (Figure 5).

In literature, it is reported that AA displays a peak corresponding to the hydroxyl group (–OH) at 3380 cm^−1^, methylene group (–CH_2_) peak at 2973 cm^−1^, carbonyl group (–C=O) peaks at 1718 and 1750 cm^−1^, –C-C group peak at 1709 cm^−1^, –C=C group peak at 1637 cm^−1^, and –C-O-C group peak at 1173 cm^−1^ [3,48,49,50]. HP-β-CD shows the –OH group band at 3414 cm^−1^, –CH group peak at 2995 cm^−1^, –C-O group peak at 1158 cm^−1^, and –C-O-C group band at 1048 cm^−1^ [51]. PVP is reported to show the –CH group stretching band at 2924 cm^−1^, –C=O group stretching peaks between 1650 and 1659 cm^−1^ [48], and –C-N group peak at 1290 cm^−1^ [3,52].

In the prepared networks, we observed the characteristic functional group peaks of individual components with little or no modification, which include appearance, shifting or disappearance of peaks, indicating the successful involvement of all components in the formed network. In blank semi-IPN hydrogels spectrum, the strong –OH group peak of HP-β-CD at 3410 cm^−1^ became weaker showing the involvement of –OH groups of HP-β-CD in prepared semi-IPN hydrogels. Small peaks at 1158 and 1048 cm^−1^ may be due to the –C-O and –C-O-C groups of HP-β-CD, respectively. In semi-IPN hydrogels, we observed an important absorption band at 937 cm^−1^ owing to the glucopyranose ring of HP-β-CD, which indicates its successful grafting onto the polymeric network as reported previously [53]. A stretching vibrational peak at 1700 cm^−1^ is due to the shifting of carbonyl group (–C=O) peaks of AA (1690 cm^−1^) and PVP (1650 and 1680 cm^−1^) in the blank hydrogels spectra showing the formation of –C-C bonding, thus showing the networking between PVP and poly(AA). The –N-H- stretching band between 3330 and 3060 cm^−1^ and –C-N stretching peak at 1650 cm^−1^ show the integration of crosslinker, i.e., MBA [3] in hydrogel matrices. Panahi et al. prepared superabsorbent semi-IPN hydrogel nanocomposites of sodium alginate-g-poly(AA)/PVP [12], and Jin et al. prepared semi-IPN hydrogels of PVP and poly(AA) [48] and found similar results as reported in this study.

Spectrum of DSP depicted the characteristic stretching vibrations of –C=O bonds at 1707, 1666, and 1624 cm^−1^ [54]. Phosphate anion of DSP shows vibrational peaks at 1299 and 1103 cm^−1^ [55]. Small vibrational peaks at 989 and 891 cm^−1^ show axial deformation of C-F group of DSP [56]. In drug-loaded semi-IPN hydrogel spectrum, one can observe all the distinguishing peaks of pure DSP from 2000 to 700 cm^−1^. In drug-loaded semi-IPN hydrogel, the phosphate anion peak of DSP merged and appeared at 1036 cm^−1^. Band of C-F group of DSP appeared at 998 cm^−1^, thus indicating the presence of DSP in the semi-IPN hydrogels. As there is little difference between the spectra of DSP-loaded and blank semi-IPN networks, we can conclude that free radical polymerization resulted in the successful formation of polymeric network and hints toward successful drug loading.

#### 2.3.2. TGA

**Figure 6:**
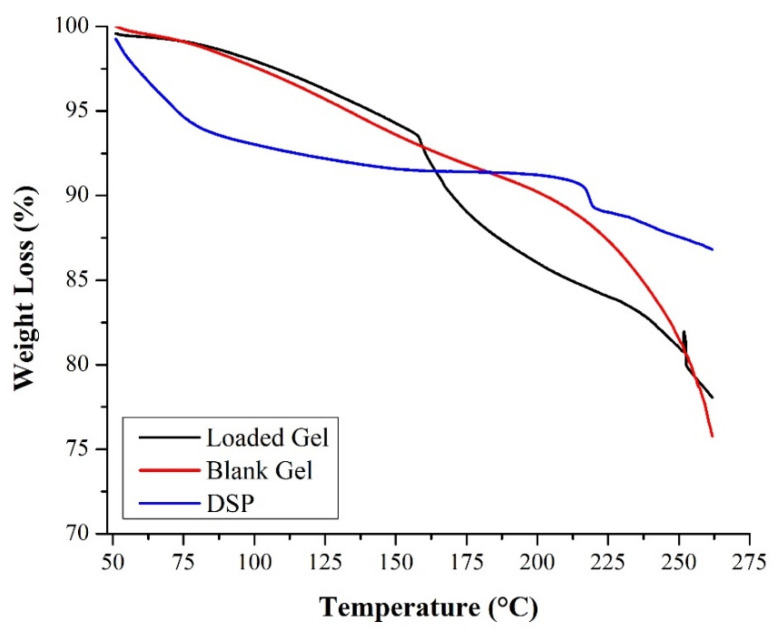
Thermogravimetric analysis of DSP, blank, and DSP-loaded semi-IPN hydrogel matrices (AP-8).

In TGA analysis, the weight change in material under testing was calculated as a function of temperature. TGA analysis is widely used to study the thermal stability and decomposition pattern of the polymers in various drug delivery systems including hydrogels. To assess the impact of thermal stress, pure drug, blank, and drug-loaded semi-IPN hydrogel matrices were subjected to thermal analysis as shown in Figure 6.

In literature, TGA curve of HP-β-CD showed two weight loss steps. The first weight loss occurred below 100 °C, which was due to water loss, and the second weight loss event occurred from 300–425 °C, which was due to the decomposition [57]. TGA curve of blank hydrogel displayed two stages, i.e., the first phase commenced from ambient temperature to 180 °C, and the second stage started from 180 °C onwards. Here, the first phase represents the evaporation of water molecules as observed in a previous study by Fujiyoshi et al. [24], which involved the development of IPN hydrogels using β-CD and N vinyl pyrrolidone (NVP). The second stage may be attributed to the degradation of PVP above 200 °C [58]. The DSP TGA curve displays different stages of weight loss (%), i.e., the first stage started from 50 to 100 °C and the second from 200 °C onwards. We attributed this weight loss to the thermal degradation of drug molecules since DSP melts at 225 °C [59].

As seen in the TGA curve of drug-loaded semi-IPN hydrogels, the results were comparable to blank hydrogels. Here, weight loss started from 150 °C, which may possibly be due to the thermal degradation of PVP and DSP, but weight loss after 225 °C was majorly due to the melting of DSP. Kamyar et al. prepared Zn/Al-CO_3_ layered double hydroxide (LDH) with various ratios of dexamethasone. TGA analysis of dexamethasone encapsulated LDH presented a mass loss between 200 and 400 °C [59]. Therefore, it is concluded that the prepared networks remained stable over a wide temperature range. Furthermore, incorporation of DSP did not affect the stability of our formulation.

#### 2.3.3. XRD

**Figure 7:**
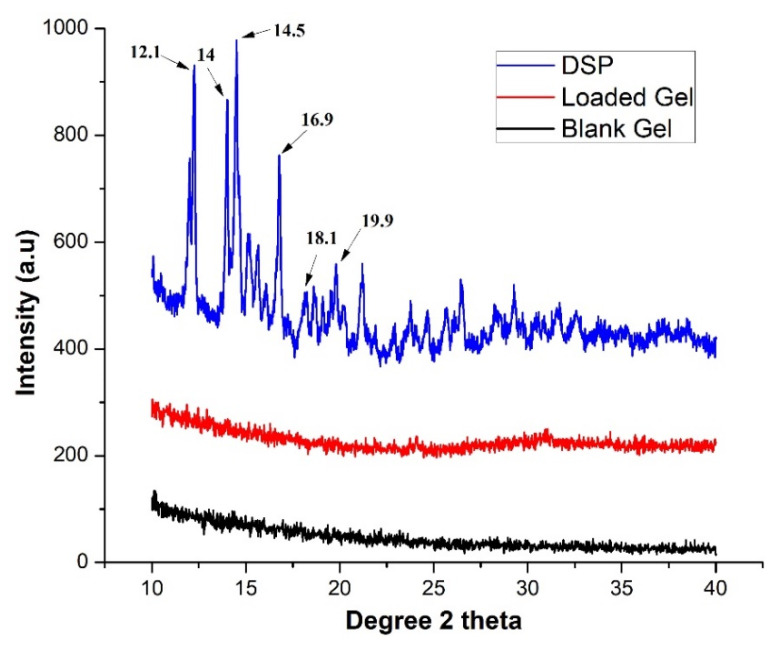
X-ray diffraction pattern of DSP, blank, and drug-loaded semi-IPN hydrogel matrices (AP-8).

XRD is employed during new drug development, manufacturing, and quality control of various dosage forms. It is a fast, nondestructive technique that provides valuable information on polymorphism, crystallinity, and amorphicity of drug molecules in solid dosage forms including semi-IPN networks. As the majority of active ingredients are often obtained as crystalline powders, scientists can use these patterns as a readily obtainable fingerprint to determine the structural type. Figure 7 shows characteristics of diffraction peaks of DSP at 12.1°, 14°, 14.5°, 16.9°, 18.1°, and 19.9°, which indicates its crystalline structure [37]. Poly(AA) is amorphous in nature [60], while the amorphous nature of HP-β-CD was shown by two broad peaks from 5–15° and 15–25° (2*θ*) [51,61].

Blank hydrogel spectrum did not show any characteristic peak and thus represented an amorphous state of hydrogels [54]. However, the reduction in crystallinity of DSP was observed in XRD spectrum of drug-loaded semi-IPN hydrogels. A slight change in behavior of semi-IPN hydrogel matrices was attributed to DSP loading as reported earlier [3]. Therefore, the amorphous nature of semi-IPN hydrogel matrices could facilitate water uptake, swelling, and drug release [62].

#### 2.3.4. SEM

**Figure 8:**
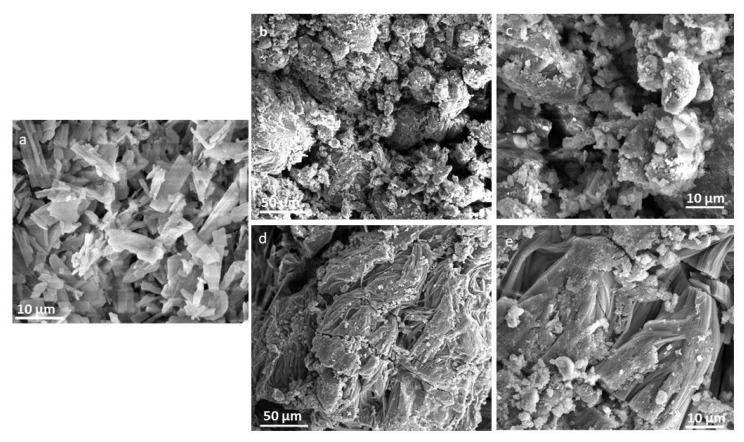
SEM of (**a**) pure drug, (**b**,**c**) blank, and (**d**,**e**) DSP-loaded hydrogel (AP-8).

This technique is extensively employed by scientists to investigate the microstructure, surface topography, and chemistry of various organic and inorganic ingredients. It provides visual information of micrometer and sub-micrometer structures of semi-IPN hydrogels. SEM pictures of DSP, blank, and DSP-loaded hydrogels (Figure 8) were obtained to observe the morphological changes in hydrogels before and after drug loading.

DSP demonstrated crystalline particles, which validates the XRD observation as discussed in the previous section. The blank semi-IPN hydrogel image shows a loose, coarse, irregular, and porous surface (Figure 8b,c). These micron size pores could arise from the interactions between PVP chains and main polymeric structure. Therefore, they can facilitate the diffusion of dissolution media into the polymeric network. Similar results were observed by Panahi et al. [12], in which the authors synthesized semi-IPN hydrogel nanocomposites of chitosan, acrylamide, AA, and PVP that were porous in nature. In another study, xanthan gum/PVP-co-poly(AA) IPN hydrogels were prepared and the hydrogels showed a rough microporous structure [9]. In the case of DSP-loaded hydrogels, a relatively smooth surface with fewer pores was observed, which might indicate the filling of pores with drugs (Figure 8d,e). Furthermore, these drug-loaded hydrogels showed small particles on their surface. These could be drug molecules, which have migrated to the surface during slow drying. These surface adhered molecules could have contributed to the initial DSP release at pH 1.2 as observed during the in vitro drug dissolution study.

### 2.4. DSP Loading and In Vitro Dissolution Study

DSP loading was executed in PBS of pH 7.4 as our gels showed maximum swelling at basic pH. Thereafter, drug release was studied in simulated gastrointestinal media using AP-8 containing 221 ± 5.40 mg of DSP per 0.3 g of gel. We observed a marked improvement in drug loading after HP-β-CD grafting, when compared to pectin-g-poly(AA)/PVP semi-IPN hydrogel, where 170.54 ± 1.75 mg of DSP was loaded in a similar-sized disc [63]. Although CDs are widely used to enhance the solubility of hydrophobic drugs even when attached to the polymeric network, they also improve the solubility of hydrophilic drugs by forming complexes with accessible hydrophobic groups or portions [27].

**Figure 9:**
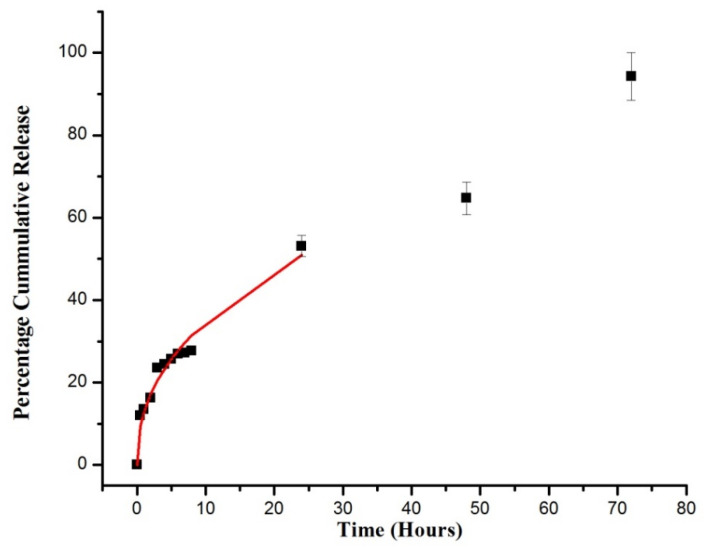
In vitro DSP dissolution from hydrogels (AP-8). Release curve shows the Korsmeyer-Peppas model on the first 60% release (shown by the red line).

Figure 9 shows the percentage cumulative DSP dissolved from the semi-IPN network at pH 1.2 for the first 2 h and then at pH 7.4 for the following 70 h. These conditions represent the pH values and transient time of GIT. An ideal colon targeted carrier should release a minimum amount of drug in the stomach but a maximum amount in the colon [64].

On estimation of the plot, we concluded that the pH had a huge impact on the DSP dissolved from the developed semi-IPN hydrogel matrices. During the first 2 h (at pH 1.2), these semi-IPNs merely released 16% of drug, thus showing an insignificant drug release in simulated gastric environment. This minute release was attributed to (a) surface-adhered drug molecules as observed in SEM image, (b) in semi-IPN network, where the –COOH groups remained intact by making hydrogen bonding with chains of semi-IPN hydrogels [37]. As a result, these hydrogels swelled slowly and released a minimum amount of the drug at pH 1.2. As the pH of media increased from 1.2 to 7.4, a large amount of the drug (94%) was released in a controlled manner. It highlights the fact that when the pH of media is above the pka of semi-IPN network, hydrogen bonds break and –COOH ionizes, which leads to the development of electrostatic repulsion and expansion of network. This leads to the increased swelling with higher drug release rate at colonic pH. These features endorse the feasibility of this formulation in treating inflammatory bowel diseases with potentially reduced side effects of DSP even after prolonged use. Corticosteroids are generally recommended for mild to moderate inflammatory bowel disease, which do not respond to aminosalicylate therapy. Systemically administered corticosteroids are reported to be less effective to maintain drug levels and thus lead to relapse, and their long-term use is associated with immune suppression, infections, diabetes, bone disease, etc. A previous study reported the preparation of pH-responsive hydrogels based on guar gum, poly(AA), and β-CD for controlled intestinal delivery of dexamethasone [40]. Our results suggest that DSP-loaded hydrogels are suitable for colonic delivery by exhibiting less than 16% of drug release at 1.2 pH and a maximum amount at 7.4 pH.

### 2.5. Drug Release Kinetics

Generally, the Korsmeyer-Peppas model is employed to analyze the drug release from drug carriers where the mechanism is not well recognized or a multiple release phenomenon is involved [3]. A value of correlation coefficient (R^2^) near “1” explains the suitability of the model, whereas “k” is the constant-associated network structure. Here, “*n*” is referred to as the diffusion exponent that elucidates the mechanisms involved in drug release. If *n* < 0.45, it denotes Fickian diffusion, 0.45 < *n* < 0.89 denotes non-Fickian diffusion, and when *n* > 0.89, it refers to the case II transport [65]. In this study, values of “*n*” and “k” were determined in the range of M_t_/M_o_ 0–60%. For AP-8, “*n*” value (Table 3) was above 0.45, which indicates that the DSP release mainly followed non-Fickian diffusion or anomalous transport where diffusion and chain relaxation are simultaneously involved. This is due to the fact that drug-loaded hydrogels are normally stored in a dry glassy state. After ingression of dissolution media, the polymeric network swells and its glass transition temperature is lowered. Concurrently, the dissolved DSP diffuses to external media through a swollen rubbery region [66]. Banarjee et al. [67] prepared controlled release IPN hydrogel microparticles of sodium carboxy methyl cellulose and poly vinyl alcohol for diclofenac sodium and reported non-Fickian drug release.

### 2.6. Hemocompatibility Study

**Figure 10:**
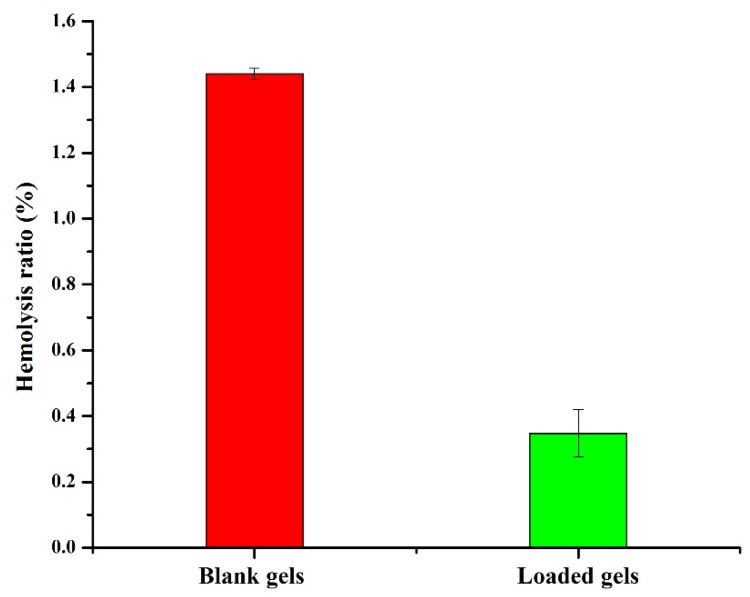
Hemocompatibility study of blank and drug-loaded hydrogels (AP-8).

Hemocompatibility test was used to assess the compatibility of the hydrogels with the biological system [68]. Hydrogels with HR above 5% are regarded as hemolytic, between 5 and 2% as slightly hemolytic, and less than 2% as non-hemolytic [69,70]. HR induced by blank and drug-loaded semi-IPN hydrogels was below 2% as shown in Figure 10. Therefore, AP-8 can be considered as non-hemolytic and can be safely used as a carrier for colon targeting. Our results are in agreement with the previously reported findings of Ghosh et al. [70], where the HR of prepared semi-IPN hydrogels of carboxy methyl guar gum and gelatin was less than 2%.

### 2.7. Toxicity Testing

#### 2.7.1. Monitoring the General Conditions of Rabbits

During acute oral toxicity testing, all clinical findings were observed prior to the study, on the 7th and 14th day of study. We did not notice any significant variation in body weight, water, and food consumption (Table 4) during this study [71]. No toxic response and deaths were observed during the 14 days of study. The animals displayed normal behavior, no signs of illness, no salivation or vomit, no diarrhea, and no dermal and ocular irritation.

#### 2.7.2. Hematological and Biochemical Analysis

Table 5 and Table 6 show that no major variations were present between hematological and biochemical profiles of the control and treated group and that they were within a normal range. Hemoglobin of both groups showed normal values. Liver and kidneys were functioning normally in both groups as observed previously [72]. These results demonstrate that the developed semi-IPN hydrogels are highly biocompatible and can be recommended for in vivo applications.

#### 2.7.3. Histopathological Evaluation

Histopathological study provides evidence for any signs of acute toxicity of drug-loaded hydrogels (Ap-8) on vital organs. For this, rabbits were sacrificed and their vital organs were excised and weighed. Weight variation was not significant among the tested groups (Table 7). In a previous study, acute toxicity of bacterial cellulose/acrylamide hydrogels was tested on mice with negligible variation in the weight of vital organs among the control and treated groups [73].

**Figure 11:**
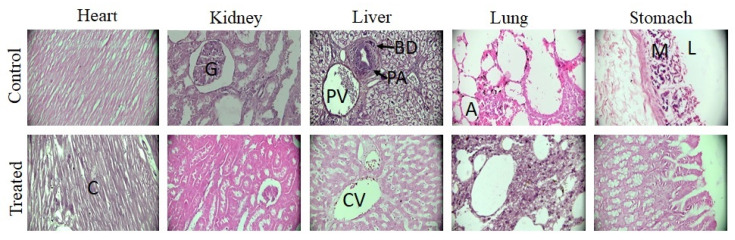
Histopathological images of vital organs of control and group treated with hydrogel after H&E staining. Here, trinocular microscope (Accu-Scope 3001) with a built-in five megapixel camera was used to take images at 40×. Alphabets in micrographs are designated as “C” for cardiomyocytes of heart, “G” for glomerulus of kidney, “CV” for central vein of liver, “PV” for portal venule, “PA” for portal arteriole, “BD” for bile duct, “A” for alveolar sacs of lung, “L” for lumen, and “M” for mucosa of stomach.

After weighing, vital organs remained in phosphate buffered neutral formalin (10% *v*/*v*). Micro sections of tissues were developed and observed under optical microscope and images were obtained. No significant changes were observed between the control and treated group (Figure 11). Heart micrographs showed that cardiac myocytes are normal, clear, arranged in good order, and nuclei were present centrally without any signs of hemorrhage or necrosis. Kidneys and liver did not show any inflammation, degradation, necrosis, and bleeding. Glomeruli showed a normal shape and portal triad was visible, while liver hepatocytes were organized into cords around the central vein [74,75]. In lungs, structures appeared normal without any collapsed alveolar sacs. Stomach micrographs showed normal stomach mucosa without any signs of ulcer. Therefore, we did not observe any noticeable pathological changes in the treated group that indicate nontoxicity and suitability of the developed formulation. Similar findings were observed previously [74], wherein gelatin-based hydrogels were prepared for colonic delivery of oxaliplatin and it was found that the prepared hydrogels were biocompatible. Furthermore, no histopathological and hematological changes were observed in rabbits. Similarly, Zhu et al. developed peptide-based bis-acrylate/AA hybrid hydrogels, which were biocompatible and non-toxic to vital organs and thus were deemed suitable for wound dressing [76].

## 3. Materials and Methods

### 3.1. Chemicals

Acrylic acid (AA), poly(vinyl pyrrolidone) K30 (PVP), and ammonium persulphate (APS) were procured from Daejung chemicals & metals Co, Ltd., Nakdong-daero, Sasang-gu, Busan, Korea. Hydroxylpropyl β cyclodextrin (HP-β-CD) was received from Roquette France as a gift sample. Ethanol and N, as well as N-methylene-bis-acrylamide (MBA) were acquired from Merck and Alfa Aesar, respectively. Remington Pharmaceutical Lahore, Pakistan gifted the dexamethasone sodium phosphate (DSP). The rest of the reagents employed were of analytical grade and used as received.

### 3.2. Fabrication of HP-β-CD-g-Poly(AA)/PVP

Free radical polymerization technique was used to develop semi-IPN hydrogels (Table 1). Initially, solutions of PVP and HP-β-CD were prepared and then mixed under constant stirring. Similarly, another solution was prepared by dissolving APS into AA and then MBA was added. The resultant solution was added to the polymeric solution under constant stirring. Nitrogen was purged for 30 min to eliminate any dissolved oxygen. Consequently, the resulting solution was decanted in glass test tubes and set in a temperature-controlled water bath at 45 °C for 1 h, 50 °C for 2 h, 55 °C for 3 h, 60 °C for 15 h, and 65 °C for 3 h. Then, hydrogel cylinders were cautiously pulled out from the test tubes after cooling to room temperature. The cylindrical hydrogels were cut into 6 mm discs, rinsed with an ethanol and water mixture (50:50 *v*/*v*) to remove traces of unreacted monomers. Thereafter, discs were dried in an oven for 24 h at 45 °C. Resultant discs were stored in airtight containers until further analysis.

### 3.3. Swelling Studies

Swelling behavior was assessed by dipping the pre-weighed dried hydrogel discs in a buffer solution of pH 1.2 or 7.4. These discs were allowed to swell in a medium until they achieved a constant weight. Surface water from the swollen discs was wiped with a filter paper and the weight was recorded after regular time intervals (0, 1, 2, 4, 6, 8, 24, 48, 72, 96, 120, 144 h) until equilibrium. Then, the swelling ratio of each formulation was determined as follows [77]:

Swelling ratio = [W_e_ − W_d_/W_d_] × 100 (1)

where W_e_ is the weight of semi-IPN hydrogel after swelling and W_d_ is the weight of dried semi-IPN hydrogel before swelling.

### 3.4. Analysis of Sol-Gel Fraction

To analyze the sol-gel fraction, freshly fabricated hydrogel cylinders were cut into 6 mm discs and dried to constant weight in an oven at 45 °C. Then, traces of non-crosslinked monomers were extracted by exposing these discs to distilled water for 48 h [78]. These extracted gels were dried to constant weight at 45 °C. Finally, the sol-gel fraction was analyzed as follows [79]:

(2) $$\%\;\mathrm{Gel}\;\mathrm{fraction}=\frac{\mathrm{Wo}}{W1}\times 100$$

% Sol fraction = 100 − Gel fraction(3)

where W_o_ is the weight of dried extracted semi-IPN hydrogels and W_1_ is the weight of non-extracted semi-IPN hydrogels after drying.

### 3.5. Drug Loading

DSP was loaded using the diffusion-assisted swelling method in optimized hydrogels, i.e., with maximum swelling. Pre-weighed dried hydrogels (AP-8) were swelled in 1% *w/w* DSP solution in PBS of pH 7.4 and maintained at 37 °C for 48 h [80]. After removal from the solution, excess surface water was blotted with a filter paper and dried to constant weight in an oven at 45 °C. The weight method was used to estimate drug loading as follows [20]:

Amount of drug = W_D_ − W_d_ (4)

Percentage drug loading was determined as follows:

(5) $$\mathrm{Drug}\;\mathrm{Loading}\;\left(\%\right)=\left[\frac{\mathrm{WD}-\mathrm{Wd}}{\mathrm{Wd}}\right]\times 100$$

where W_D_ is the weight of dried semi-IPN hydrogels after loading and W_d_ is the weight of dried semi-IPN hydrogels before loading.

### 3.6. Solid-State Characterization

FTIR was used to elucidate possible chemical structures and interactions of DSP with hydrogel matrices and were scanned in the range of 4000–500 cm^−1^. SDT Q 600 TA Universal was used to acquire TGA curves of DSP, blank, and drug-loaded semi-IPN hydrogel matrices. About 5 mg of the test sample was heated up to 300 °C at a heating rate of 10 °C/min under a constant stream of nitrogen in sealed aluminum pans. X’pert PRO, PANalytical, Netherlands was used to record XRD patterns of DSP, blank, and DSP-loaded semi-IPN hydrogel matrices. Finally, the morphology of DSP, blank, and DSP-loaded HP-β-CD-g-poly(AA)/PVP semi-IPN hydrogels was observed by SEM. Hydrogels were sputter-coated with gold and examined using FEI Quanta 250 SEM (Hillsboro, OR, USA) at different resolutions [81,82].

### 3.7. In Vitro DSP Dissolution

DSP dissolution from the optimized semi-IPN hydrogel (AP-8) was determined using the paddle apparatus (Tianjin Guoming Medicinal Equipment Co. Ltd. Tianjin, China), maintained at 37 ± 0.5 °C with a paddle rotation set at 50 rpm. Each hydrogel disc was immersed in a vessel containing 0.1 N HCl (500 mL with pH 1.2) for 2 h to create simulated gastric conditions and then transferred to PBS (500 mL with pH 7.4) for the next 70 h to create simulated intestinal conditions. At suitable intervals, 5 mL of dissolution media was sampled and immediately replaced with a similar volume of fresh media to maintain sink conditions. Collected samples were filtered (0.45 μm syringe filters) and the filtrate was analyzed at 242 nm by spectrophotometry (Agilent, Model 8453). For each run, the percent cumulative drug dissolved was determined by Equation (6) [83]. The experiment was repeated in triplicate, and the average was obtained to draw the release curve as follows:

(6) $$\%\;\mathrm{drug}\;\mathrm{release}\;=\frac{\mathrm{Mt}}{\mathrm{Mn}}\times 100$$

where M_t_ represents the amount of drug released at any given time “t” and M_n_ signifies the amount of drug loaded in semi-IPN hydrogel matrices. M_t_ was obtained by placing absorbance values in a calibration curve equation (y = 0.026x + 0.004). After drug release studies, the Korsmeyer-Peppas model was used to assess the drug release mechanism as shown [82] below:

M_t_/M_o_ = k_KP_ t^n^ (7)

where M_t_ is the amount of drug released in time “t”, M_o_ is the quantity of DSP released at infinity, K_KP_ is the release rate constant, and *n* is the diffusional coefficient.

### 3.8. Hemocompatibility Study

Hemocompatibility test was performed on the optimized semi-IPN hydrogel formulation (AP-8) by a minor adjustment of the previously reported method [70]. Briefly, hydrogel disc was dipped in isotonic saline solution (0.9% NaCl) and equilibrated at 37 °C for 24 h. Then, 2 mL of anticoagulated human blood (with EDTA 1% *w/v*) was obtained from healthy donors and the hydrogel sample with 5 mL of PBS (7.4 pH) was added to the test sample. Blood with buffer or distilled water remained in screw-capped tubes as negative and positive controls, respectively. Then, these tubes were incubated for 60 min at 37 °C. Thereafter, the sample was centrifuged at 2500 rpm for 20 min, and the absorbance of supernatant was recorded at 575 nm by UV visible spectrophotometer (Agilent, Model 8453). The test was performed in triplicate and the hemolysis ratio (%) was recorded as follows [84]:

(8) $$\mathrm{Hemolysis}\;\mathrm{ratio}\;(\mathrm{HR})\;\left(\%\right)=\frac{\mathrm{AS}\;-\;\mathrm{ANC}\;}{\mathrm{APC}\;-\;\mathrm{ANC}\;}\;\times \;100$$

where A_S_ is the absorbance of test sample, A_NC_ is the absorbance of negative control, and A_PC_ is the absorbance of positive control.

### 3.9. Toxicity Testing

This study was conducted according to the fixed dose guideline number 420, set by The Organization for Economic Co-Operation and Development (OECD). All protocols were approved by the Ethical Committee of GCUF vide letter number GCUF/ERC/2153. Herein, we obtained twelve rabbits from an in-house facility and organized them into two groups. Here, Group I (control) only received water and food, while Group II (treated group) received 2 g/kg of DSP-loaded semi-IPN hydrogels (AP-8) [85]. These animals were carefully observed for any physical changes, mortality rate, body weight changes, water and food consumption during the period of study. After 14 days, a blood sample was obtained for assessment of hematological and biochemical parameters, and the rabbits were sacrificed to obtain vital organs. These organs were fixed in 10% (*v*/*v*) phosphate buffered neutral formalin and stained with hematoxylin and eosin (H&E) for histopathological analysis [9].

## 4. Conclusions

HP-β-CD-g-poly(AA)/PVP semi-IPN hydrogel matrices were successfully developed by employing the free radical polymerization technique. Herein, we found that the formulation (AP-8) with PVP (1.33%) and AA (16.66%) was mechanically strong, elegant in appearance, and pH-responsive with excellent swelling at pH 7.4. The structure and morphology of HP-β-CD-g-poly(AA)/PVP networks were probed by FTIR and SEM, respectively. FTIR results confirmed the accomplishment of polymerization reaction and the absence of interaction between DSP and semi-IPN hydrogels, while SEM results indicated the rough porous surface and crystalline nature of DSP, which was further confirmed by XRD. The optimized hydrogel formulation (AP-8) restricted the DSP release at pH 1.2, while providing the controlled delivery of DSP at colonic pH over an extended time. Release kinetics revealed the non-Fickian diffusion mechanism for DSP release. The prepared semi-IPN hydrogels were hemocompatible and nontoxic and thus can be considered safe for biological systems. Based on our findings, with the ever-increasing demand for new biocompatible materials for tissue repair and drug delivery, we conclude that the fabricated semi-IPN network has potential for colonic delivery of DSP to treat inflammatory bowel diseases. Moreover, in future, the semi-IPN network could be equally used for other biomedical applications.

## Figures and Tables

**Table 1:** Ingredients of HP-β-CD-g-poly(AA)/PVP semi-IPN hydrogel matrices.

Trial Code	AA g/100 g	PVP g/100 g	Formulation Code	AA g/100 g	PVP g/100 g	Formulation Code	AA g/100 g	PVP g/100 g
AP-1 *	0	0.3	AP-7 *	0	1.33	AP-13 *	0	2.66
AP-2	16.66		AP-8	16.66		AP-14	16.66	
AP-3	33.33		AP-9	33.33		AP-15	33.33	
AP-4	50		AP-10	50		AP-16	50	
AP-5	66.66		AP-11	66.66		AP-17	66.66	
AP-6	83.33		AP-12	83.33		AP-18 +	83.33	

* No gel formed, + Gel burst. HP-β-CD and MBA remained constant, i.e., 0.3 g/100 g, while APS was 0.16 g/100 g in all formulations.

**Table 2:** Sol-gel fraction of semi-IPN hydrogel matrices (*n* = 3).

Sr No	Trial Code	Gel (%)	Sol (%)	Code	Gel (%)	Sol (%)	Code	Gel (%)	Sol (%)
1	AP-1 *	-	-	AP-7 *	-	-	AP-13 *	-	-
2	AP-2	95.68	4.31	AP-8	99.59	0.40	AP-14	96.66	3.33
3	AP-3	92.41	7.58	AP-9	93.48	6.51	AP-15	92.45	7.54
4	AP-4	92.37	7.62	AP-10	93.41	6.58	AP-16	90.27	9.72
5	AP-5	94.21	5.78	AP-11	93.33	6.66	AP-17	89.88	10.11
6	AP-6	97.23	2.76	AP-12	93.28	6.71	AP-18 +	-	-

* No gel formed, + Gel burst.

**Table 3:** DSP dissolution kinetics from AP-8 using the Korsmeyer-Peppas model.

Model	Parameter	Value
Korsmeyer-Peppas	Release rate constant (K)	11.799
	Diffusion exponent (*n*)	0.472
	Correlation coefficient (R^2^)	0.9800

**Table 4:** Clinical findings of all groups (*n* = 6).

Parameters	Control	Treated
Signs of illness	None	None
Dermal toxicity	None	None
Ocular toxicity	None	None
Mortality rate	None	None
Body weight (kg)		
Pretreatment	1.36 ± 0.05	1.33 ± 0.05
1st day	1.33 ± 0.11	1.30 ± 0.1
7th day	1.23 ± 0.11	1.34 ± 0.23
14th day	1.33 ± 0.15	1.36 ± 0.15
Water Intake (mL)		
Pretreatment	183.66 ± 3.05	192 ± 2.64
1st day	181.33 ± 3.21	186 ± 1.73
7th day	185.66 ± 2.08	190 ± 1.73
14th day	189 ± 1.73	193 ± 1.52
Food Intake (g)		
Pretreatment	65.55 ± 1.52	60.33 ± 1.52
1st day	68 ± 2.64	58.33 ± 1.52
7th day	65.3 ± 3.05	63 ± 1
14th day	68 ± 1.73	65.66 ± 1.15

**Table 5:** Biochemical analysis of blood.

Hematology	Control	Treated
Hemoglobin (10–15 g/dL)	12.8 ± 0.55	12.6 ± 0.05
TLC (4.5–11 × 109 L^−1^)	3.86 ± 0.56	3.7 ± 0.3
Red Blood Cells (4.2–5.9 × 1012 L^−1^)	5.55 ± 0.22	5.67 ± 0.01
Platelets (150–400 × 109 L^−1^)	159 ± 01	358 ± 13.11
Monocytes (2–8%)	3.66 ± 0.57	3.66 ± 0.57
Neutrophils (40–60%)	52 ± 1	20 ± 4
Lymphocytes (20–40%)	78.3 ± 0.57	79 ± 1
Eosinophils (1–4%)	2.33 ± 0.57	2 ± 1
Mean Corpuscular Volume (80–96 fL)	62.96 ± 0.55	61.16 ± 1.4
Mean Corpuscular Hemoglobin (27–32 pg)	22.4 ± 0.62	23.36 ± 0.97
Mean Corpuscular Hemoglobin Concentration (32–36%)	34.9 ± 0.26	38.26 ± 0.90

**Table 6:** Liver, kidney, and lipid profiles of control and treated group.

Biochemical Analysis	Control	Treated
Liver profile		
Alanine aminotransferase (17–77 IU/L)	91 ± 1	37 ± 3
Aspartate aminotransferase (54–298 IU/L)	114.33 ± 1.52	53 ± 2.64
Renal profile		
Creatinine (0.2–0.9 mg/dL)	0.56 ± 0.04	0.62 ± 0.16
Urea (10–50 mg/dL)	16 ± 2	19 ± 3
Uric acid (3.4–7.1 mg/dL)	3.8 ± 0.1	4.93 ± 0.80
Lipid profile		
Cholesterol (10–80 mg/dL)	66.48 ± 1.15	68.3 ± 2.08
Triglycerides (46–68 mg/dL)	54.73 ± 1.48	54.3 ± 0.57

**Table 7:** Weight variation of vital organs after oral administration of hydrogels. All values are expressed in the standard deviation (*n* = 6).

Group	Heart (g)	Kidney (g)	Liver (g)	Lung (g)	Stomach (g)
Control	3.68 ± 0.07	9.18 ± 1.52	35.01 ± 1.99	16.56 ± 2.65	12.25 ± 1.93
Treated	3.06 ± 0.25	6.90 ± 0.29	28.48 ± 1.01	7.39 ± 0.46	8.42 ± 0.97

## Data Availability

The majority of data are presented in the article. Raw or processed data required to reproduce these findings cannot be shared at this time due to technical or time limitations.
